# Facile Strategy for the Synthesis of Gold@Silica Hybrid Nanoparticles with Controlled Porosity and Janus Morphology

**DOI:** 10.3390/nano9030348

**Published:** 2019-03-03

**Authors:** Marina Santana Vega, Andrés Guerrero Martínez, Fabio Cucinotta

**Affiliations:** 1 School of Natural and Environmental Sciences, Newcastle University, Newcastle upon Tyne NE1 7RU, UK; M.Santana-Vega2@newcastle.ac.uk; 2 Departamento de Química Física, Universidad Complutense de Madrid, Avenida Complutense s/n, 28040 Madrid, Spain

**Keywords:** mesoporous silica, gold nanoparticles, Janus nanoparticles, sol–gel synthesis, MCM-41

## Abstract

Hybrid materials prepared by encapsulation of plasmonic nanoparticles in porous silica systems are of increasing interest due to their high chemical stability and applications in optics, catalysis and biological sensing. Particularly promising is the possibility of obtaining gold@silica nanoparticles (Au@SiO_2_ NPs) with Janus morphology, as the induced anisotropy can be further exploited to achieve selectivity and directionality in physical interactions and chemical reactivity. However, current methods to realise such systems rely on the use of complex procedures based on binary solvent mixtures and varying concentrations of precursors and reaction conditions, with reproducibility limited to specific Au@SiO_2_ NP types. Here, we report a simple one-pot protocol leading to controlled crystallinity, pore order, monodispersity, and position of gold nanoparticles (AuNPs) within mesoporous silica by the simple addition of a small amount of sodium silicate. Using a fully water-based strategy and constant content of synthetic precursors, cetyl trimethylammonium bromide (CTAB) and tetraethyl orthosilicate (TEOS), we prepared a series of four silica systems: (A) without added silicate, (B) with added silicate, (C) with AuNPs and without added silicate, and (D) with AuNPs and with added silicate. The obtained samples were characterised by transmission electron microscopy (TEM), small angle X-ray scattering (SAXS), and UV-visible spectroscopy, and kinetic studies were carried out by monitoring the growth of the silica samples at different stages of the reaction: 1, 10, 15, 30 and 120 min. The analysis shows that the addition of sodium silicate in system B induces slower MCM-41 nanoparticle (MCM-41 NP) growth, with consequent higher crystallinity and better-defined hexagonal columnar porosity than those in system A. When the synthesis was carried out in the presence of CTAB-capped AuNPs, two different outcomes were obtained: without added silicate, isotropic mesoporous silica with AuNPs located at the centre and radial pore order (C), whereas the addition of silicate produced Janus-type Au@SiO_2_ NPs (D) in the form of MCM-41 and AuNPs positioned at the silica–water interface. Our method was nicely reproducible with gold nanospheres of different sizes (10, 30, and 68 nm diameter) and gold nanorods (55 × 19 nm), proving to be the simplest and most versatile method to date for the realisation of Janus-type systems based on MCM-41-coated plasmonic nanoparticles.

## 1. Introduction

The encapsulation of plasmonic nanosystems in silica structures, especially gold nanoparticles (AuNPs), has attracted tremendous attention over the last two decades [1,2] since the thermal and mechanical robustness and optical transparency of this material make it an ideal candidate to expand the applications of metal nanoparticles. The Stöber synthesis [3] of dense silica has been, for the most part, the approach chosen by researchers to improve the colloidal [4], chemical, [5] and thermal [6] stability of metal nanoparticles. However, recent developments have allowed the incorporation of AuNPs into mesoporous materials, such as MCM-41, whose pores can host multiple organic and inorganic guests, thus, broadening their application range to catalysis [7], cell imaging [8] and drug delivery [9,10,11]. More recently, great efforts have been devoted to the development of methodologies to generate anisotropy within these systems by means of asymmetric growth of silica shells, opening the way to a new generation of Janus mesoporous NPs [12]. 

Janus NPs are named after the ancient Roman god of duality, frequently depicted as having two faces, the same way Janus NPs present two different chemical or physical properties on opposite locations. The potential applications of such structures arise from their unique interfacial activity and anisotropy, which allows directional interactions and selective reactivity, attributes traditionally observed in molecules. This expands enormously the applications of traditional isotropic NPs to a wide range of fields, such as biomedicine and sensing [13,14,15,16] or selective catalysis [17,18], while they can also be employed as emulsion stabilisers [19], surfactants [20], optical probes [21] and displays [22]. The latest approaches to synthesise this type of structure can be classified into masking, self-assembly and phase separation methods [23], allowing the incorporation of organic and inorganic materials with different properties. Recent strategies for the incorporation of metals with specific functionalities make use of a modification of the templating surfactant structure and have been employed for the preparation of aluminosilicate nanoparticles [24,25]. Special attention has also been given to the design of gold@silica composites (hereafter Au@SiO_2_ NPs) with different shapes. The incorporation of AuNPs into silica shells to generate such systems was first achieved by functionalisation of their surface using a binary mixture of ligands, generating a vitreophylic face over which silica grows [26,27,28,29,30]. Janus Au@SiO_2_ NPs can also be prepared by following a synthetic route that allows the growth of mesoporous instead of amorphous silica by using alcohols as solvents or co-solvents and controlling the addition rate of the precursor tetraethyl orthosilicate (TEOS) [31,32,33,34], by realising Pickering emulsions using melted paraffin wax [12,35], or by anchoring functionalised AuNPs to a glass or silicon substrate over which the silica grows [36]. 

We intended to develop a simpler approach to prepare Janus Au@SiO_2_ NPs using a fully water-based procedure and minimising the number of reagents and synthetic steps. For this purpose, we looked at the basic routes employed for the synthesis of a family of mesoporous materials discovered in 1992 [37] by researchers at the Mobil Research and Development Corporation called M14S, which includes three different discrete structures: MCM-48 (cubic), MCM-50 (lamellar), and MCM-41 (hexagonal). This discovery expanded the potential of mesoporous materials enormously, since it broke the pore-size constraints of microporous zeolites (<15 Å), providing materials with extremely high surface areas (>1000 m^2^ g^−1^) and still well-defined pore sizes (15–100 Å) and pore order. Owing to such properties, mesoporous silica materials have been widely investigated over the last 20 years [38], not only for the previously mentioned thermal stability and optical transparency, but also for their straightforward surface functionalisation under mild reaction conditions, opening up new application routes in drug delivery [39], light harvesting [40], or catalysis [41], to mention a few.

During the 1990s, most researchers used sodium silicate as the inorganic precursor [42,43,44,45,46,47] and an alkyl ammonium surfactant, typically cetyl trimethylammonium bromide or chloride (CTAB or CTAC, respectively), for the synthesis of M14S structures. Silicate salts, however, are not very reactive in water (even under alkaline or acidic conditions); therefore, the synthesis requires hydrothermal conditions, e.g., high temperatures and pressures and long reaction times. To circumvent these issues, alkoxysilanes (in particular TEOS) have become the most commonly used precursors, as they present higher hydrolysis and condensation rates in water and can produce ordered materials at room temperature and pressure [48,49,50]. Despite the high reactivity of TEOS in alkaline solution, it takes several days to achieve highly ordered materials at room temperature. Radu et al. solved this problem by heating the reaction mixture at 80 °C, reducing the reaction time from several hours or even days to only 2 h [51]. To date, this is the most effective method and the fact that the template for this type of silica is usually based on micelles of CTAB or CTAC makes it a perfect candidate for the incorporation of AuNPs, since their colloidal synthesis in such surfactants has been extensively investigated, and several methodologies have been reported to produce monodisperse AuNPs of different shapes and sizes [52]. 

As mentioned above, both traditional silica-coated and Janus NPs have been reported using MCM-41 [12,31,32,33,34,35,36]. However, the previous studies employed methodologies that rely on the use of binary solvent mixtures and varied concentrations of CTAB and silica sources, with reproducibility limited to specific nanoparticle (MCM-41 NP) sizes. Therefore, it would be highly beneficial to develop a simplified method based on the use of one solvent only (preferably water), the variation of the least number of synthesis parameters (e.g., reactants concentrations) and the possibility to adapt it to different types of AuNPs.

To develop such an approach, it is of key importance to focus on the mechanism of MCM-41 nanoparticle (MCM-41 NP) growth and its determining factors. Researchers at Mobil proposed a liquid crystal templating (LCT) mechanism [37], in which the surfactant molecules pack into a hexagonal array before the addition of the inorganic silica source. However, the concentration of surfactant typically used for the synthesis of MCM-41 is vastly insufficient to form hexagonal arrays, and so the LCT mechanism in its literal sense is only possible under specific synthetic conditions [53]. Another mechanism was postulated by cooperative self-assembly of the alkyl ammonium surfactant micelles and silicate precursor species. Several groups have demonstrated the efficacy of this mechanism, as supported by NMR and small angle X-ray scattering (SAXS) studies [54]. Among them, Monier et al. proposed a charge density matching mechanistic model that suggested the existence of a lamellar mesophase formed from electrostatic interactions between the surfactant molecules and silicate species [55], which would transition from lamellar to hexagonal to maintain the charge density balance with the surfactant head groups as the silicate species begin to condense. More recently, the development of time-resolved synchrotron SAXS has been crucial to monitor in situ the growth of MCM-41 [56], which revealed a unique insight into the evolution of the micelle structure upon addition of silicate species. In this regard, Yi et al. proposed a swelling–shrinking model that postulates that the micelles self-assemble as positively charged ellipsoids into which TEOS is solubilised, making them transition into spheres [57]. Upon TEOS hydrolysis, the silica monomers become hydrophilic and are expelled from the hydrophobic cores of the micelles, condensing on their surface and making them shrink. According to this model, the addition of a second source of silica with different solubility, in this case, sodium silicate, may have a great impact on the micelle structure during the first stages of the synthesis, which would strongly affect the crystallinity of the final product. This is because the silicate salts are soluble in water and charged in alkaline solution; thus, they may interact with the positively charged head groups of the surfactant molecules before the hydrolysis of TEOS begins. In light of this effect, a method to manipulate the direction of the silica growth and its crystallinity simply by controlling the amount of silicate would be of enormous help, being further exploited to confer specific directionality to the synthesis of hybrid Au@SiO_2_ NPs.

Here, we report a simple one-pot reaction methodology that not only increases the crystallinity of the MCM-41 but also allows control over the position of the AuNPs within the mesoporous silica nanostructures upon addition of a small amount of sodium silicate, offering the possibility of realising Janus NPs based on mesoporous silica-coated AuNPs.

## 2. Materials and Methods

### 2.1. Characterisation Techniques

UV-visible measurements: Localised surface plasmon resonance (LSPR) bands were measured by absorption spectroscopy. The spectra were recorded on a Hitachi U3310 spectrophotometer (Newcastle upon Tyne, UK) and a BioTek Instruments Uvikon XL UV-Visible spectrophotometer (Madrid, Spain). All experiments were carried out at 298 K using 1 cm path length quartz cuvettes. The obtained values of the LSPR bands are reported for each type of AuNPs in Section 2.2.1. 

Transmission electron microscopy (TEM): TEM pictures were obtained on a JEOL JEM 1400 microscope (Madrid, Spain) at an acceleration voltage of 120 kV. TEM grid preparation: typically, 5 to 10 mg of silica nanoparticles were dispersed in 2 mL of milli-Q water, using an ultrasonic bath. Then, ca. 20 mL of such dispersions were deposited on a 400 square mesh copper grid and allowed to air dry. From the obtained micrographs, average size and distribution of the synthesised Au@SiO_2_ nanoparticles and AuNPs were obtained, by measuring the diameter of at least 100 particles using ImageJ software.

Small angle X-ray scattering (SAXS): SAXS diffractograms were obtained using an X-ray generator ANalytical PW3830 and a Hecus-Braun camera (Madrid, Spain).

### 2.2. Synthesis

All starting materials and chemicals were obtained from commercially available sources and used without further purification. CTAB (≥98%), TEOS (98%) and l-Ascorbic Acid (≥99.0%) were purchased from Sigma-Aldrich (Haverhill, UK); Sodium Silicate solution (d = 1.5 g/mL) was purchased from Fisher Scientific (Loughborough, UK); Sodium Borohydride (98%) was purchased from Alfa Aesar (Heysham, UK); CTAC (≥98.0%), Gold(III) chloride trihydrate (≥99.9%), 5-Bromosalicylic acid (90%) and Sodium hypochlorite solution (available chlorine 10−15%) were purchased from Aldrich (Haverhill, UK), and Sodium hydroxide (98%) was purchased from Quimipur (Madrid, Spain). In all the following procedures, high purity water was used, with a conductivity of 0.04 mS cm^−1^, obtained from a Milli-Q purification system (Newcastle upon Tyne, UK). 

#### 2.2.1. Synthesis of AuNPs

Gold nanospheres (AuNSs): Single-crystal AuNSs of different sizes were synthesised following a seed-mediated protocol previously described elsewhere [58], with minor modifications. For the preparation of the gold seeds, 5 mL of 0.1 M CTAC (0.5 mmol, 0.16 g) was mixed with 50 μL of a 0.05 M HAuCl_4_ solution (2.5 × 10^−3^ mmol, 0.98 mg) and 25 µL of ascorbic acid 0.1 M (2.5 × 10^−3^ mmol, 0.44 mg). Immediately after the addition of ascorbic acid, 200 µL of a freshly prepared 0.02 M NaBH_4_ solution (4 × 10^−3^ mmol, 0.15 mg) was injected under vigorous stirring. The seed solution presented a brown/yellowish colour that turned darker after 3 min of aging. Solutions presenting shades of pink or red colour were discarded.

Ten nanometres AuNSs: 50 mL of a 25 mM CTAC solution (1.25 mmol, 0.4 g) was mixed with 200 µL of 0.1 M ascorbic acid (2.5 × 10^−3^ mmol, 3.52 mg) and 450 μL of the seed solution. Then, 250 μL of a 0.05 M HAuCl_4_ solution (1.25 × 10^−2^ mmol, 4.92 mg) was injected under vigorous stirring. The mixture was left undisturbed at room temperature until the solution presented a bright red colour (for at least 10 min). The resulting AuNSs presented an LSPR band centred at 522 nm, and the diameter of the AuNSs was 10 ± 1 nm.

Thirty nanometres AuNSs: 300 mL of a 25 mM CTAC solution (7.5 mmol, 2.4 g) was mixed with 1.2 mL of ascorbic acid (0.12 mmol, 21.13 mg) and 3 mL of the 10 nm sphere solution. Then, 1.5 mL of a 0.05 M HAuCl_4_ (7.5 × 10^−2^ mmol, 29.54 mg) solution was injected under vigorous stirring. The mixture was left undisturbed at room temperature until the solution presented a bright red colour (for at least 20 min). The resulting AuNSs presented an LSPR band centred at 538 nm. The AuNSs were etched by adding 300 µL of a dilute sodium hypochlorite solution (1 to 1.5 wt% of available chlorine). The solution was stirred at 30 °C for 15 min, then centrifuged (6500 rpm for 30 min) and redispersed in CTAB several times. After the last cycle, the concentration was adjusted to [CTAB] = 5.3 mM, [Au^0^] = 0.76 mM (3.4 × 10^11^ AuNS/mL). The LSPR band after oxidative etching was centred at 527 nm and the diameter of the AuNSs was 31 ± 1 nm.

Sixty-eight nanometres AuNSs: 300 mL of a 25 mM CTAC solution (7.5 mmol, 2.4 g) was mixed with 1.2 mL of ascorbic acid (0.12 mmol, 21.13 mg) and 0.3 mL of the 10 nm sphere solution. Then, 1.5 mL of a 0.05 M HAuCl_4_ (7.5 × 10^−2^ mmol, 29.54 mg) solution was injected under vigorous stirring. The mixture was left undisturbed at room temperature until the solution presented brown colour (at least 30 min). The resulting AuNSs presented an LSPR band centred at 562 nm. The AuNSs were etched by adding 300 µL of a dilute sodium hypochlorite solution (1–1.5 wt% of available chlorine), followed by 115 μL of 0.05 M HAuCl_4_. The solution was stirred at 30 °C for 150 min, then centrifuged (3500 rpm for 30 min) and redispersed in CTAB several times. After the last cycle, the concentration was adjusted to [CTAB] = 5.3 mM and [Au^0^] = 5.52 mM (3.4 × 10^11^ AuNS/mL). The LSPR band after oxidative etching was centred at 537 nm and the diameter of the AuNSs was 68 ± 4 nm.

Gold nanorods (AuNRs): Single-crystal AuNRs with an average length of 55 ± 5 nm, diameter of 19 ± 1 nm and aspect ratio of 2.90 ± 0.15 were synthesised by a seeded growth method with minor modifications [59]. The seeds were prepared by the standard CTAB/NaBH_4_ procedure: 25 µL of a 0.05 M HAuCl_4_ solution (1.25 × 10^−3^ mmol, 0.49 mg) was added to 4.7 mL of a 0.1 M CTAB solution (4.7 × 10^−1^ mmol, 171.2 mg); 300 µL of a freshly prepared 0.01 M NaBH_4_ solution (3 × 10^−3^ mmol, 0.11 mg) was then injected under vigorous stirring. For the synthesis of AuNRs, 45 mg of 5-bromosalicylic acid (0.2 mmol) was added to 50 mL of 0.05 M CTAB (2.5 mmol, 0.91 g) and the mixture was mildly stirred for 15 min until complete dissolution. Then, 480 µL of 0.01 M AgNO_3_ (4.8 × 10^−3^ mmol, 0.82 mg), 500 µL of 0.05 M HAuCl_4_ (2.5 × 10^−2^ mmol, 9.84 mg) and 200 µL of a 0.1 M ascorbic acid solution (2 × 10^−2^ mmol, 3.52 mg) were added to the mixture. After 2 h at 25 °C, 50 µL of 0.1 M ascorbic acid (5 × 10^−3^ mmol, 0.88 mg) and 80 µL of the seed solution were added under vigorous stirring. After 2 h, 5 µL of 0.1 M ascorbic acid was added to complete the reduction process. The mixture was left undisturbed at room temperature for at least 4 h. Then, the AuNRs were washed three times for 40 min (8000 rpm, 30 °C) and redispersed in 5.3 mM CTAB. The transversal and longitudinal LSPR bands were centred at 509 nm and 722 nm, respectively.

#### 2.2.2. Synthesis of MCM-41 NPs

The synthesis of all silica samples was reproduced from a previous publication [51]. Typically, 6 mL of 5.3 mM CTAB (3.18 × 10^−2^ mmol, 11.6 mg) was placed in a 20 mL round-bottomed flask and connected to a reflux condenser. The temperature was raised to 80 °C, and 43 µL of 2 M NaOH (8.6 × 10^−2^ mmol, 3.44 mg) was added. The solution was stirred for a few minutes before dropwise addition of 60 µL of TEOS. The mixture was stirred at 80 °C for 2 h. After this time, the mixture was allowed to cool to room temperature and the silica was filtered or centrifuged (10 min at 3000 rpm) and washed with water several times (except in the case of the samples prepared for the kinetic studies, which were cooled to 0 °C in an ice bath, then centrifuged and left to air dry without further washing). For the samples containing sodium silicate, 40 μL of a diluted (1:10) sodium silicate solution was added together with the NaOH.

#### 2.2.3. Synthesis of Au@SiO_2_ NPs

**Scheme 1:**
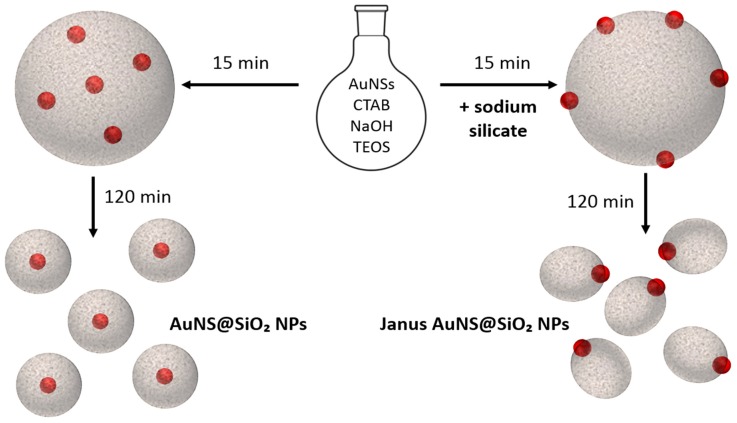
Scheme illustrating the synthetic strategy used for the synthesis of gold@silica nanoparticles (AuNS@SiO_2_ NPs).

The AuNPs (6 mL, 3 × 10^11^ AuNP/mL) in 5.3 mM CTAB (Table 1) were placed in a 20 mL round-bottomed flask, and the reaction was carried out as described in Section 2.2.3 (Scheme 1).

## 3. Results and Discussion

### 3.1. Mechanistic Studies on MCM-41 NP Growth

**Figure 1:**
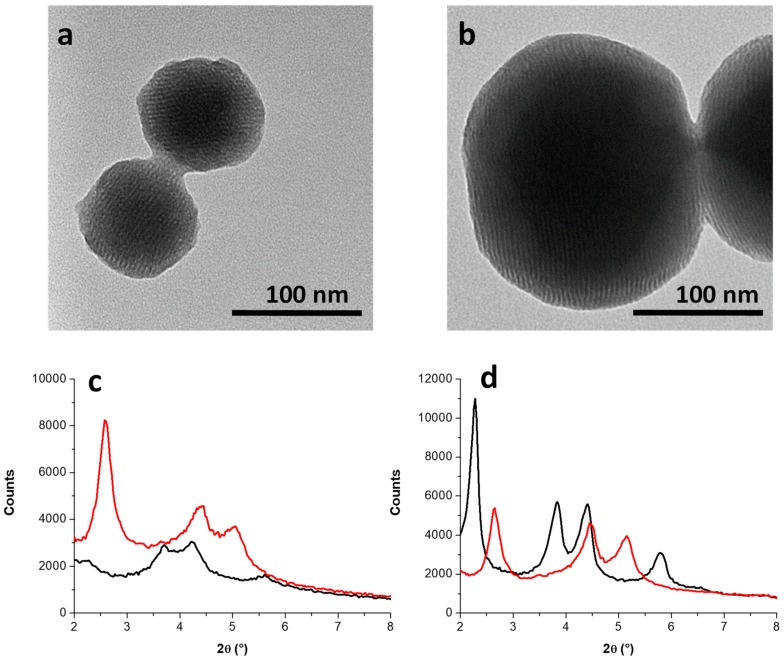
Top: Transmission electron microscopy (TEM) micrographs of MCM-41 nanoparticle (MCM-41 NPs) synthesised following: (**a**) route A and (**b**) route B (addition of sodium silicate); scale bar = 100 nm. Bottom: Small angle X-ray scattering (SAXS) diffractograms of MCM-41 NPs for (**c**) system A and (**d**) system B, before (black) and after (red) calcination.

All samples were prepared following the protocol (Table 1) described by Radu et al. mentioned above [51]. To a CTAB solution heated to 80 °C, sodium hydroxide and TEOS were added (systems A and B), together with a small amount of sodium silicate (system B only). After 2 h of reflux, the samples obtained did not present the same grade of crystallinity. Figure 1a,b show the TEM images of MCM-41 NPs synthesised in the absence and presence of sodium silicate. It is clear from these images that the pore order is vastly improved upon addition of sodium silicate. The samples prepared which reproduced Radu’s protocol (system A) were very sensitive to the addition rate of TEOS and the scale of the reaction. Since this synthesis is performed at 80 °C, whereas previous protocols based on TEOS are carried out at room temperature and with longer reaction times [48,49,50], this loss of crystallinity may be explained in terms of a less ordered surfactant template. An increase of the critical micelle concentration (CMC) due to a higher motion of the surfactant molecules could be the cause of this loss of order. However, the CMC of CTAB has been reported to not be strongly affected in the 25 to 80 °C temperature range [60]. Furthermore, system B was also synthesised at 80 °C and afforded, in all cases, highly crystalline materials regardless of the reaction scale and TEOS addition rate. Thus, our hypothesis is that the lower crystallinity of system A with respect to system B is due to a faster hydrolysis rate of TEOS, which would result in less-controlled condensation of the silica framework. This was also confirmed by SAXS (Figure 1c,d): the diffractogram reflections at 2*θ* = 2.58, 4.40, and 5.10° can be indexed, respectively, to the (100), (110), and (200) planes of a hexagonal pore array. Nevertheless, among the various research groups employing the high-temperature protocol described for system A (no sodium silicate addition), some reported highly crystalline samples [61,62,63,64,65,66]. Silicate salts are a common impurity in sodium hydroxide pellets [66], and we deem their presence to be one of the possible causes of the capricious nature of this method. To rule out any influence of silicates in our experiments, we confirmed the purity of the sodium hydroxide in our lab by elemental analysis. Indeed, the amount of silicon in our pellets was 252 μg/g, which is below the minimum content of sodium silicate necessary to observe the above effect.

In the case of system A before calcination, the (100) reflection at 2*θ* = 2.25° (*d* = 39.2 Å) is barely visible. This is due to a large excess of surfactant in the system as the silica samples were not washed after the reaction to observe the water-soluble intermediate, as explained in the next section. However, after calcination, it is possible to observe this reflection at 2*θ* = 2.58° (*d* = 34.1 Å) with higher intensity. The shift to greater 2*θ* values, also observed in system B, corresponds to a pore contraction typically observed when MCM-41 NPs are exposed to high temperatures (550–600 °C) owing to the removal of the surfactant template and partial condensation of the silanol groups on the internal surface of the pores [67,68].

#### 3.1.1. Kinetic Study on MCM-41 in the Absence/Presence of Sodium Silicate

**Figure 2:**
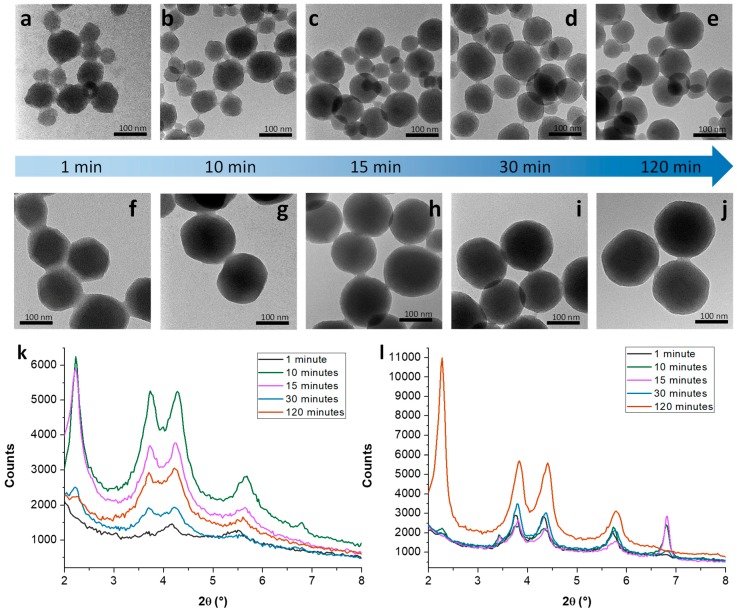
Top: TEM micrographs of MCM-41 NPs at different stages of the synthesis, following route A (**a**–**e**) and B (**f**–**j**); scale bar = 100 nm. (**a**–**e**): System A at 1, 10, 15, 30 and 120 min, respectively; (**f**–**j**) system B at 1, 10, 15, 30 and 120 min, respectively. Bottom: SAXS diffractograms of MCM-41 NPs at different reaction stages from system A (**k**) and system B (**l**).

The two routes of synthesis of systems A and B, following the protocol mentioned above, were monitored by TEM and SAXS. Aliquots of both reactions were taken at 1, 10, 15, 30 and 120 min and quickly cooled to 0 °C before centrifugation to monitor the growth of the MCM-41 NPs as accurately as possible by suppressing it. Figure 2 shows the TEM images and SAXS diffractograms of the aliquots, while Table 2 summarises the MCM-41 NP size distribution for each of them. 

System A: The first solid silica was formed after 1 min. The channels are already visible from the TEM images (Figure 2a–e), but the SAXS diffractogram shows only weak reflections corresponding to the formation of a hexagonal array due to a CTAB excess (the samples were not washed after the reaction was stopped) (Figure 2k). Nevertheless, the peaks at 2*θ* values of 2.2°, 3.8° and 4.3°, corresponding, respectively, to the (100), (110) and (200) reflections, are intense enough to indicate the formation of a hexagonal array, even at this early stage of the synthesis. The maximum crystallinity was achieved after only 10 min when the three reflections are the most intense of all the aliquots. After this time, there is an apparent loss of crystallinity as the reaction continues. The reflection at 2*θ* = 6.8° (*d* = 13 Å) corresponds to a lamellar mesophase [69] formed upon interaction of the silanol groups of the growing silica oligomers and the surfactant molecules, as discussed in Section 3.1.2.

System B: When the reaction was performed in the presence of sodium silicate, the reflections corresponding to the hexagonal array were more intense than those in system A at all reaction times (Figure 2l), even after the first minute, although maximum crystallinity was not achieved until 120 min of reaction. This means that, although the hexagonal array seems to be formed quicker than in system A, the overall reaction time is significantly longer. In addition to said longer reaction time, reflections at 2*θ* = 6.8° and 3.4° corresponding to a lamellar phase are visible from the first measurement; such phase coexists in equilibrium with the hexagonal phase for the first 10 min. After this time, both reflections begin to lose intensity and are no longer visible after 30 min. As can be seen from Table 2, system B produces bigger NPs than system A, with a generally higher degree of pore order (Figure 2e,j).

In the presence of silicates, the NPs are also more stable through the reaction, as shown by the constant trend of increasing order with the reaction time and present higher monodispersity as well (Figure 2f–j). The NPs synthesised in the absence of silicates present a spherical shape with irregular edges, while the addition of sodium silicate produces more defined NPs with an elongated shape and, in some cases, it is even possible to identify a rounded hexagonal shape.

#### 3.1.2. Growth Mechanism of MCM-41 NPs in the Absence/Presence of Sodium Silicate

In both systems (A and B), when the hydrolysis of TEOS begins, the negatively charged –SiO^−^ groups interact with the positive head groups of the CTAB molecules, generating lamellar aggregates that form crystals when the reaction is cooled to 0 °C [69]. The lamellar phase appears at the same time as the hexagonal phase but disappears as the reaction proceeds and the hexagonal phase develops. The disappearance of the reflections at 2*θ* = 3.4 and 6.8° indicates the completion of the reaction, which occurs between 10 and 15 min in system A, and between 30 min and 2 h in the case of system B (Figure 2). This indicates that the addition of silicate salts reduces the growth rate of the MCM-41 NPs, leading to improved crystallinity of the system. Unlike TEOS, silicate salts are soluble in water and negatively charged when they are added to the reaction mixture, which may have two different effects: first, the silicate salts interact strongly with the polar groups of the micelles containing TEOS [57], thus, slowing down the release of TEOS to the hydrophilic medium where it hydrolyses; second, the equilibrium of the hydrolysis reaction may be influenced by the presence of these silicates, as they may compete with the –SiO^−^ groups from TEOS that form before the condensation step takes place [70]. Based on these observations and previous mechanistic studies, we propose the following growth mechanism:

Before the addition of TEOS, the concentration of CTAB is above the CMC, so the CTAB molecules form spherical (or ellipsoidal) micelles. In the case of system B, a strong electrostatic interaction occurs between the negatively charged silicate ions and the positive polar heads of the CTAB micelles. At this stage, TEOS is added to the reaction mixture, and silica growth begins. TEOS solubilises in the CTAB micelles, since it is not soluble in the hydrophilic medium, but eventually hydrolyses, gaining a net negative charge. Consequently, hydrolysed TEOS becomes water soluble and leaves the micelle core, interacting with the micelle surface. During this process, a lamellar intermediate forms as a result of the electrostatic interactions between the –SiO^−^ groups and polar CTAB heads. This lamellar phase transitions into a hexagonal phase as the condensation of TEOS proceeds, maintaining the charge balance. When the reaction is performed in the absence of silicate salts (system A), the electrostatic interactions around the micelles are weaker, TEOS molecules are more easily released from the micelles, and they start to condense faster as a result of the high temperature. This leads to the rapid disappearance of the lamellar phase within the first few minutes of the reaction, and the hexagonal phase is formed indicating the end of the reaction. On the other hand, when silicate salts are present (system B), they may not participate in the condensation phase owing to their relative lower content ([Na_2_SiO_3_]/[TEOS] = 1/20) and reactivity compared to those of TEOS. Therefore, they likely remain negatively charged throughout the whole reaction, maintaining the strong interactions between the micelles and, thus, stabilising the lamellar intermediate and extending its lifetime. Eventually, all TEOS molecules hydrolyse and condense to form a silica framework, and the lamellar phase slowly reorganises into an ordered hexagonal phase, resulting in highly crystalline mesoporous silica particles (Figure 2). 

This mechanism also explains why the MCM-41 NPs produced in system B, that is, in the presence of sodium silicate, are bigger and have a better-defined shape than the ones produced in system A (Figure 2 and Table 2). Since the amount of TEOS is kept constant in both reaction systems, the larger NPs observed in system B can only mean a smaller amount of them than in system A. The slower silica growth rate in system B makes the –SiO^−^ groups diffuse at a faster rate than they react. This gives them more time to rearrange through diffusion around existing nucleation seeds and continue the MCM-41 NP growth, instead of condensing randomly in the solution and generating more nucleation seeds and, therefore, more NPs. A slower growth rate not only implies fewer nucleation seeds being formed but also a longer living intermediate lamellar phase. Such a phase forms at the early stages of the reaction (up to 15–30 min, see diffractograms in Figure 2), when not all TEOS has reacted via condensation. As the reaction progresses, the lamellar phase grows and eventually collapses into a cylindrical phase, since this geometrical shape offers a larger surface, over which to spread the charges more effectively [55,56]. The cylindrical micelles then self-assemble rapidly to form a hexagonal mesophase, producing better defined MCM-41 NPs than those obtained in system A.

### 3.2. Incorporation of AuNPs in Mesoporous Silica

**Figure 3:**
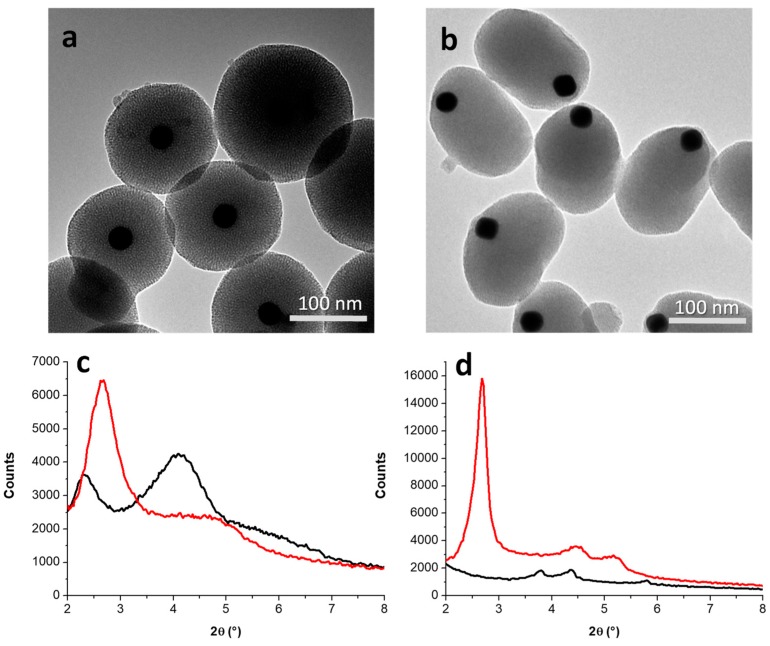
Top: TEM micrographs of gold@silica nanoparticles (AuNS@SiO_2_ NPs) with AuNSs incorporated: (**a**) at the centre, with radial mesopores (system C), and (**b**) at the edge, with parallel mesopores (system D); scale bar = 100 nm. Bottom: SAXS diffractograms for (**c**) system C and (**d**) system D, before (black) and after calcination (red).

The addition of sodium silicate to the traditional MCM-41 synthesis with TEOS not only slows down the silica growth and improves the crystallinity of the MCM-41 NPs but can also be employed to control the mesoporosity and the position of AuNPs within the silica shell. As can be seen from Figure 3a, the traditional synthesis of MCM-41 incorporating AuNSs (30 nm of diameter) in the reaction mixture (Table 1) results in radially oriented pores and AuNSs placed at the centre of the isotropic NPs (system C). Indeed, the diffractogram in Figure 3c, recorded after calcination of the sample, confirms the deviation of the pore order from the hexagonal pattern to a less regular radial: the diffraction peaks assignable to the (110) and (200) planes of the hexagonal array become ambiguous or disappeared, and the peak corresponding to the (100) plane is broadened, indicating disordering of the mesopores [71]. On the other hand, the addition of sodium silicate to the reaction (system D) results in a completely different organisation, with MCM-41-like channels and AuNSs placed at the edge of the NPs (Figure 3b,d). At such experimental conditions, no significant differences in the MCM-41 NP diameter were observed with respect to system B (Table 2). This means that, following a very simple methodology, Janus AuNS@SiO_2_ NPs can be synthesised. This methodology can also be used to incorporate AuNPs of different shapes and sizes both at the centre and at the edges of NPs with a high degree of reproducibility and control over the loading and position of the AuNPs (as described in Section 3.3).

#### 3.2.1. Kinetic Study on AuNS@SiO_2_ NPs in the Absence/Presence of Sodium Silicate

**Figure 4:**
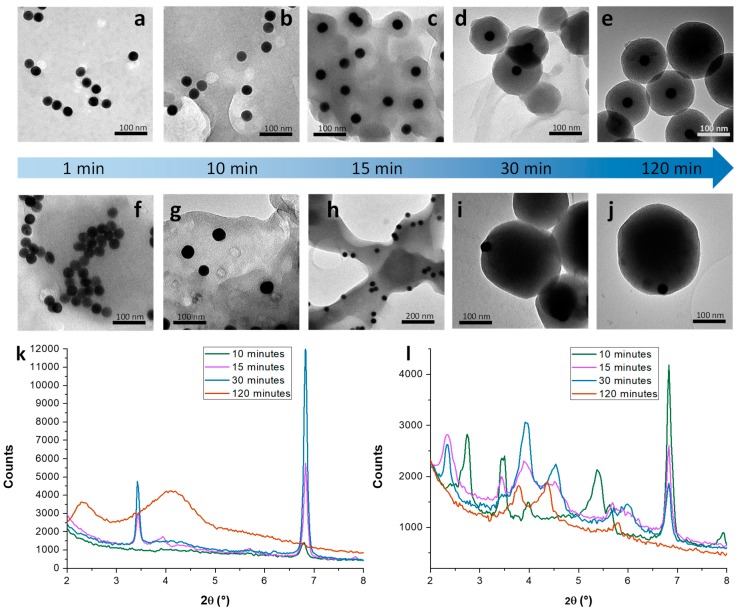
Top: TEM micrographs of AuNS@SiO_2_ NPs at different stages of the synthesis, following route C (**a**–**e**) and D (**f**–**j**); scale bar = 100 nm, except for picture h (scale bar = 200 nm). (**a**–**e**) System C at 1, 10, 15, 30 and 120 min, respectively; (**f**–**j**) system D at 1, 10, 15, 30 and 120 min, respectively. Bottom: SAXS diffractograms at different reaction stages for system C (**k**) and system D (**l**).

Two samples were prepared under the same conditions (Table 1), incorporating AuNSs of 30 nm in diameter, with the only difference being the absence (system C) or addition (system D) of a small amount of sodium silicate. Aliquots of both reactions were taken at 1, 10, 15, 30 and 120 min and cooled to 0 °C before centrifugation to more accurately monitor the growth of the mesoporous silica layer. Figure 4 shows the TEM images and SAXS diffractograms of the aliquots.

System C: After 1 min (Figure 4a), the Au NSs show a thin layer of silica and cannot be isolated as a solid. This silica seems to be amorphous. After 10 min (Figure 4b), the AuNSs are embedded in the bulk of amorphous silica, showing only one Bragg reflection at 2*θ* = 6.8° (*d* = 13 Å) (Figure 4k), corresponding to the surfactant/silica lamellar agglomerate discussed in the previous section. This bulk silica starts to dissociate into spherical silica NPs 5 min later (Figure 4c–e), but the diffractogram does not show any sign of pore order yet. The reflection at 2*θ* = 3.4° (*d* = 26 Å) comes from the agglomerate mentioned above, and the process continues until all the isotropic AuNS@SiO_2_ NPs are apart from each other. Interestingly, the pores do not seem to form until the end of the reaction, where two broad signals in the SAXS diffractogram are observed (Figure 4k), indicating a radial distribution of the pores.

System D: As observed in system C, a thin layer of silica is formed after 1 min, but no solids are obtained up to 10 min (Figure 4f,g). In this case, the bulk silica shows some degree of pore order. The reflections corresponding to the (100), (110), and (200) planes typically attributed to hexagonal arrangements shift to lower 2*θ* values (higher *d*-spacing values) as the reaction goes on, indicating that the pores expand during the synthesis (Figure 4l). Indeed, the (100) reflection is not visible after 120 min, unless the sample is calcined. The lamellar phase is also present in equilibrium with the hexagonal phase. The TEM picture at 15 min of reaction shows how the AuNSs are no longer distributed randomly across the silica bulk (Figure 4h) but have a preference for the silica–medium interface. This is the key point of the mechanism since this distribution places the AuNSs at the edge and not at the centre of the MCM-41 NPs at the end of the reaction (Figure 4i,j). 

#### 3.2.2. Growth Mechanism of AuNS@SiO_2_ NPs in the Absence/Presence of Sodium Silicate

The radial pore distribution observed in system C has been widely reported [7]. The AuNSs capped with CTAB molecules act as nucleation sites for the growth of mesoporous silica. Traditionally, it has been accepted that these CTAB molecules form a bilayer surrounding the AuNPs [72]; however, recent computational calculations [73] have demonstrated that the energy required for such molecular cohesion is not compensated by the entropy loss necessary to stretch the hydrocarbon tails within the layer, making the system evolve into a heterogeneous distribution of adsorbed micelles. When the hydrolysis and condensation of TEOS begins, there are two competitive nucleation sites: the adsorbed CTAB micelles surrounding the AuNPs and the free CTAB micelles that reorganise upon the addition of TEOS, as it occurs in a traditional MCM-41 synthesis. In the case of system C, it is obvious that the predominant process is the nucleation of silica on the surface of the AuNSs. As can be seen from Figure 4, during the first seconds after the addition of TEOS, a thin silica layer is formed around the AuNSs. Then, the silica starts to condensate encapsulating the AuNSs into a large amorphous silica gel, which finally disaggregates into spherical AuNS@SiO_2_ NPs. On the other hand, upon addition of sodium silicate (system D), strong interactions are established between the silicate ions and CTAB polar heads, similarly as those in system B. This process causes the nucleation to occur preferably on the free CTAB micelles, leading to the disappearance of the lamellar intermediate in favour of the hexagonal phase and allowing a parallel distribution of the channels, which are formed in the early stages of the synthesis. Indeed, parallel channels were observed in the gel formed after 10 min (Figure 4h), and the hexagonal arrangement was confirmed by SAXS (Figure 4l). Figure 4i shows how the AuNSs remain preferably at the interface between the silica gel and the medium as the NPs detach from the growing gel. This results in the AuNSs being placed at the edge of the finally formed MCM-41 NPs, which maintain a parallel distribution of the pores.

### 3.3. Incorporation of AuNPs of Different Sizes and Shapes in Mesoporous Silica

**Figure 5:**
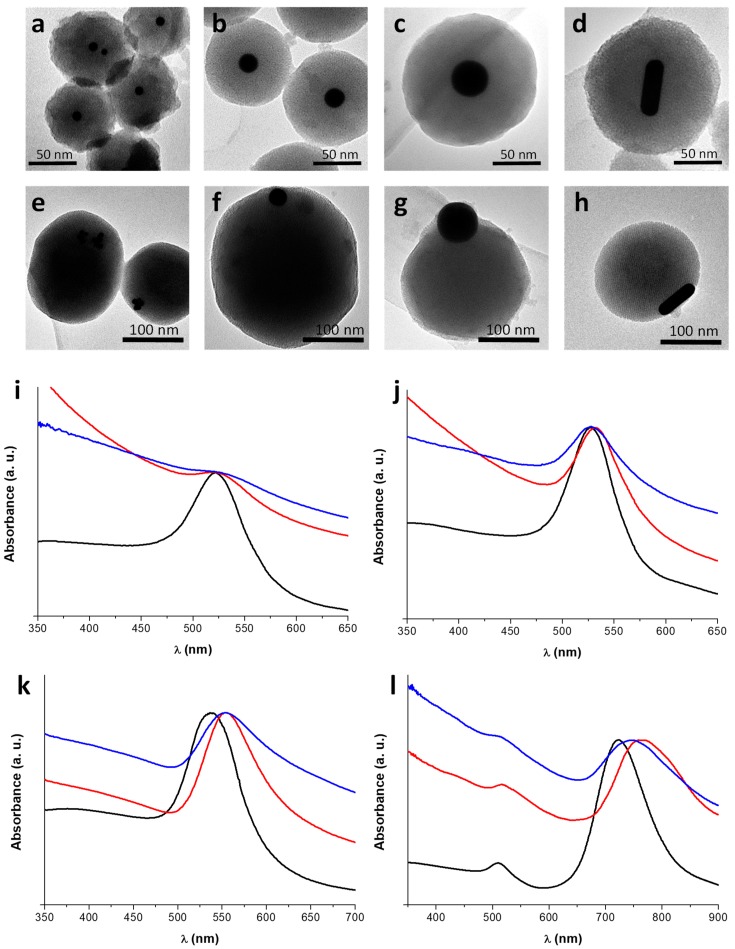
Top: TEM micrographs of isotropic Au@SiO_2_ NPs (**a**–**d**) and Janus Au@SiO_2_ NPs (**e**–**h**), using: (**a**,**e**) Gold nanospheres (AuNSs) of 10 nm, **b**,**f**) AuNSs of 30 nm, **c**,**g**) AuNSs of 68 nm and (**d**,**h**) Gold nanorods (AuNRs); scale bars = 50 nm (**a**–**d**) and 100 nm (**e**–**h**). Bottom: UV-visible spectra of gold nanoparticles (AuNPs) (black), isotropic Au@SiO_2_ NPs (red) and Janus Au@SiO_2_ NPs (blue), using: (**i**) AuNSs of 10 nm, (**j**) AuNSs of 30 nm, (**k**) AuNSs of 68 nm, and (**l**) AuNRs.

The same methodology employed for the synthesis of systems C and D was used to produce silica NPs incorporating AuNSs of different diameters (10, 30 and 68 nm) and AuNRs (55 × 19 nm) (Table 1). The position of the AuNPs within the silica shell could be controlled by addition of the same amount of sodium silicate to the reaction mixture as that employed for system D. The synthesis was carried out maintaining the number of AuNPs constant, as described in the experimental section. Figure 5 shows the TEM pictures of these Au@SiO_2_ NPs, as well as their UV-visible spectra to track any changes in the LSPR bands.

The optical characterisation shows, in all cases, a red-shift of the LSPR band upon encapsulation of the AuNPs within the silica shells. This is due to the higher refractive index of silica compared to that of water [6]. Interestingly, the LSPR band shift of the Janus Au@SiO_2_ NPs is smaller than that of the isotropic Au@SiO_2_ NPs, since the AuNPs are closer to the silica–water interface, therefore, less influenced by the silica medium (Table 3). These anisotropic samples also present higher scattering due to their larger size (Table 3). In the case of the isotropic AuNS@SiO_2_ NPs, this shift is more pronounced as the diameter increases due to the characteristic higher intensity and sensitivity of the LSPR generated by larger particles [58]. Additionally, the longitudinal LSPR band of AuNRs is known to be more sensitive, which explains why this shift is much more accentuated for their systems [73]. 

It can be observed from Figure 3a-h and Table 3 that the size of the isotropic Au@SiO_2_ NPs incorporating AuNPs at their centre depends on the size of the AuNPs, while the Janus Au@SiO_2_ NPs present similar sizes. This reinforces our initial hypothesis: in the absence of sodium silicate, the AuNPs act as nucleation sites for silica growth and, therefore, the final hybrid NP size is determined by the dimensions of the nucleus. On the other hand, when the AuNPs are placed at the edge of the mesostructure, the final NP size is not strongly influenced by the AuNP diameter. However, such hybrid NPs are still larger than those synthesised without AuNPs (system B, Table 2).

## 4. Conclusions

Mesoporous silica-coated AuNPs were successfully synthesised through a simple strategy that enables control over the crystallinity, pore order and position of the AuNPs. Using a fully water-based sol–gel route and by adding a controlled small amount of sodium silicate to the traditional reagent set used for MCM-41 silica, we demonstrated that it is possible to obtain highly crystalline Janus systems. Kinetic studies by TEM and SAXS analyses at different stages of the formation of the Au@SiO_2_ NPs hybrids showed that the effect of silicate addition is essential in promoting slow silica growth and imparting a highly ordered hexagonal pore arrangement, with the AuNPs selectively confined at the silica–water interface. In the absence of sodium silicate, the Au@SiO_2_ NPs hybrids adopt an isotropic organisation, with the AuNPs encapsulated in the centre of the system exhibiting radial pore order. Our methodology has been applied to AuNPs of different spherical sizes (10, 30, and 68 nm) and also to rods, producing the same results and, thus, demonstrating the wide versatility of such a simple strategy, which can potentially be extended to any type of metallic nanoparticles, providing a powerful tool for the control of directionality and reactivity in plasmonic nanomaterials.

## Figures and Tables

**Table 1:** Details of the synthesis of different types of MCM-41 nanoparticle (MCM-41 NPs) and gold@silica nanoparticles (Au@SiO_2_ NPs).

Sample	Sodium Silicate	[Au^0^], mol L^−1^/10^−4^ (AuNP Size)
System A	NO	-
System B	YES	-
System C	NO	7.6 (30 nm)
System D	YES	7.6 (30 nm)
AuNS@SiO_2_ NPs (10 nm)	NO	1.6 (10 nm)
Janus AuNS@SiO_2_ NPs (10 nm)	YES	1.6 (10 nm)
AuNS@SiO_2_ NPs (30 nm)	NO	7.6 (30 nm)
Janus AuNS@SiO_2_ NPs (30 nm)	YES	7.6 (30 nm)
AuNS@SiO_2_ NPs (68 nm)	NO	55.2 (68 nm)
Janus AuNS@SiO_2_ NPs (68 nm)	YES	55.2 (68 nm)
AuNR@SiO_2_ NPs	NO	7.9 (55 × 19 nm)
Janus AuNR@SiO_2_ NPs	YES	7.9 (55 × 19 nm)

**Table 2:** Average diameter (nm) of MCM-41 NPs synthesised *via* routes A and B.

	1 min	10 min	15 min	30 min	120 min
System A	73 ± 20	76 ± 22	84 ± 25	81 ± 20	81 ± 21
System B	127 ± 31	148 ± 26	145 ± 28	159 ± 22	157 ± 17

**Table 3:** Average dimension of Au@SiO_2_ NPs incorporating gold nanoparticles (AuNPs) of different sizes and shapes; optical shift of Au@SiO_2_ NPs with respect to the localised surface plasmon resonance (LSPR) wavelength maximum of the AuNPs (see experimental).

Sample	Diameter (nm)	LSPR Wavelength (nm)	AuNPs LSPR Wavelength (nm)	LSPR Shift (nm)
AuNS@SiO_2_ NPs (10 nm)	99 ± 11	523	522	1
Janus AuNS@SiO_2_ NPs (10 nm)	203 ± 51	523	522	1
AuNS@SiO_2_ NPs (30 nm)	153 ± 15	531	527	4
Janus AuNS@SiO_2_ NPs (30 nm)	215 ± 34	528	527	1
AuNS@SiO_2_ NPs (68 nm)	207 ± 15	554	537	17
Janus AuNS@SiO_2_ NPs (68 nm)	221 ± 39	554	537	17
AuNR@SiO_2_ NPs	145 ± 10	745	(509), 722	23
Janus AuNR@SiO_2_ NPs	231 ± 35	765	(509), 722	43
